# Insulin regulation of solute carrier family 2 member 1 (glucose transporter 1) expression and glucose uptake in decidualizing human endometrial stromal cells: an in vitro study

**DOI:** 10.1186/s12958-020-00674-0

**Published:** 2020-11-21

**Authors:** Ivika Jakson, Dorina Ujvari, Sebastian Brusell Gidlöf, Angelica Lindén Hirschberg

**Affiliations:** 1 Department of Women’s and Children’s Health, Karolinska Institutet, Karolinska vägen 37A, 171 76 Stockholm, Sweden; 2 Women’s Health Theme, Karolinska University Hospital, Stockholm, Sweden; 3 Department of Clinical Science, Intervention and Technology, Karolinska University Hospital, Stockholm, Sweden; 4 Department of Obstetrics & Gynecology, Stockholm South General Hospital, Stockholm, Sweden; 5 Department of Gynecology and Reproductive Medicine, Karolinska University Hospital, Stockholm, Sweden

**Keywords:** Decidualization, GLUT1, SLC2A1, Insulin, Glucose uptake, Human endometrium

## Abstract

**Background:**

Solute carrier family 2 member 1 (SLC2A1; previously known as glucose transporter 1), is the most abundant glucose transporter in human endometrium and is up-regulated during decidualization, whereas high insulin may have a negative impact on this process. The present study aimed to investigate the effect of insulin on the expression of SLC2A1 and glucose uptake in decidualizing human endometrial stromal cells.

**Methods:**

We induced in vitro decidualization of endometrial stromal cells obtained from regularly menstruating healthy non-obese women. The cells were treated with increasing concentrations of insulin, and the involvement of the transcription factor forkhead box O1 (FOXO1) was evaluated using a FOXO1 inhibitor. *SLC2A1* mRNA levels were measured by Real-Time PCR and protein levels were evaluated by immunocytochemistry. Glucose uptake was estimated by an assay quantifying the cellular uptake of radioactive glucose. One-way ANOVA, Dunnett’s multiple comparisons test and paired t-test were used to determine the statistical significance of the results.

**Results:**

We found that insulin dose-dependently decreased *SLC2A1* mRNA levels and decreased protein levels of SLC2A1 in decidualizing human endometrial stromal cells. Transcriptional inactivation of *FOXO1* seems to explain at least partly the down-regulation of *SLC2A1* by insulin. Glucose uptake increased upon decidualization, whereas insulin treatment resulted in a slight inhibition of the glucose uptake, although not significant for all insulin concentrations.

**Conclusions:**

These results indicate an impairment of decidualization by high concentrations of insulin. Future studies will determine the clinical significance of our results for endometrial function and decidualization in women with insulin resistance and hyperinsulinemia.

## Background

Decidualization is a progesterone-induced extensive remodeling of the endometrium in preparation for a potential pregnancy. The extensive remodeling includes morphological transformation of spindle-shaped endometrial stromal cells into decidual cells characterized by increased size and circularity, specific subcellular composition with more ribosomes, lysosomes, glycogen and lipids, and comprehensive reprogramming of the gene expression profile [1, 2]. Improper decidual transformation is proposed to lead to various pregnancy complications, like impaired implantation, recurrent pregnancy loss and preeclampsia [1, 3, 4].

During decidualization, stromal cells show increasing levels of glycogen indicating a glucose-dependent process [5]. Glucose transporters are membrane proteins responsible for glucose uptake in the cells. Their tissue distribution depends on metabolic activity and energy needs of the tissue. At least seven isoforms have been identified in human endometrial stromal cells (hESC). Solute carrier family 2 member 1 (SLC2A1; previously known as glucose transporter 1) is the most abundant glucose transporter in the endometrial stromal cells [6]. In contrast to solute carrier family 2 member 4 (SLC2A4; previously known as GLUT4) that relocates from intracellular vesicles to the plasma membrane only upon insulin signaling, SLC2A1 is classically a non-insulin-responsive glucose transporter responsible for the constitutive transport of glucose. However, insulin has been shown to regulate expression and to some extent cellular location of SLC2A1 in different tissues [7–9].

SLC2A1 protein levels increase up to ten times during decidualization. This increase in SLC2A1 is accompanied by an increase in cellular glucose uptake [10, 11]. On the other hand, both knockdown of *SLC2A1* and in vitro decidualization under low-glucose conditions are associated with decreased decidual marker expression [6, 12]. Together, these data suggest a functional role of SLC2A1 in decidualization.

We have previously demonstrated that insulin affects the expression of many decidual markers via transcriptional and post-translational inhibition of forkhead box O1 (*FOXO1*), an important transcription factor during decidualization [13]. Our study suggested that high concentrations of insulin may have a negative impact on the comprehensive reprogramming of gene expression during decidualization, However, morphological transformation was not inhibited by insulin. These results could be clinically relevant to hyperinsulinemic conditions like polycystic ovary syndrome (PCOS) and obesity associated with endometrial dysfunction, reduced fertility, recurrent pregnancy loss, preeclampsia and gestational diabetes [14, 15]**.** In support, we showed that weight reduction in obese women with PCOS, resulted in enhanced endometrial insulin signaling and increased SLC2A1 expression [16]. This observation led us to the hypothesis that insulin might regulate SLC2A1 during decidualization of human endometrial stromal cells.

There is lack of knowledge about how insulin and SLC2A1 might interact in the glucose-dependent process of decidualization. Further studies of such interaction could increase our understanding of underlying mechanisms for reduced fertility and pregnancy complications in disorders of hyperinsulinemia. The main objective of the present in vitro study was to investigate the effect of different doses of insulin on the gene and protein expression of SLC2A1 in relation to glucose uptake in decidualizing human endometrial stromal cells. Furthermore, we aimed to study if SLC2A1 is regulated by *FOXO1*.

## Methods

### Subjects

The subjects were six healthy women aged 21–33 with regular menstrual cycles, body mass index 22–28 kg/m^2^, no hormonal treatment 3 months prior to examination, no chronic disease, non-smoking and no regular medication. On cycle day 5–9, an endometrial biopsy was collected using an endometrial suction curette (Pipet Curet, CooperSurgical, USA).

### Isolation of endometrial stromal cells

Endometrial stromal cells were isolated immediately after collection using a protocol previously described [13]. Sequential culturing and immunocytochemistry with cytokeratin and CD10 staining ensured the purity of the obtained stromal cells.

### Culture conditions

We used 6-well Costar plates (Sigma-Aldrich, USA) for all experiments except immunocytochemistry where Falcon chambered cell culture slides (Thermo Fischer Scientific, USA) were used. hESC were cultured until 70–80% confluence in a culture media consisting of DMEM/F12-Glutamax (Thermo Fischer Scientific, USA), 10% fetal bovine serum (Thermo Fischer Scientific, USA) and 0.2% penicillin-streptomicin (Thermo Fischer Scientific, USA). Cells were then decidualized in phenol-red free DMEM/F12 (Thermo Fischer Scientific, USA) supplemented with 2% charcoal stripped fetal bovine serum (Sigma-Aldrich, USA) and 0.2% penicillin-streptomycin for 5–6 days (depending on the experiment) using 1 μM medroxyprogesterone-17-acetate (MPA) (Sigma-Aldrich, USA) and 0.5 mM N^6^, 2`-O-dibutyryladenosine cAMP (db-cAMP) (Sigma-Aldrich, USA) in the presence or absence of 5, 50 or 500 nM insulin (Sigma-Aldrich, USA). The insulin concentrations were chosen based on previous reports of tissue concentrations of insulin in different organs being significantly higher than circulating levels. In the experiment with FOXO1 inhibitor, cells were pre-decidualized for 3 days before further treatment with decidualizing agents in the presence of 100 nM and 500 nM AS1842856 (FOXO1-inhibitor by Merck Millipore, Germany, dissolved in DMSO) for 2 more days. The culture media was changed every 3 days during culturing and treatments.

### RNA isolation, cDNA synthesis and RT-PCR

RNA isolation and cDNA synthesis were done as previously described [16]. The *SLC2A1* gene expression level was quantified with Real-Time PCR using TaqMan Assay (Assay ID Hs00892681_m1 for *SLC2A1* and Hs01926559_g1 for ribosomal protein L13a as an internal control). All determinations were performed in triplicate and the relative gene expression levels were determined using the ΔΔC_T_ method.

### Immunocytochemistry

Cells were treated with 1 μM MPA and 0.5 mM N^6^ db-cAMP in the presence or absence of 500 nM insulin. After treatment, the cells were fixed with 4% paraformaldehyde for 15 min and kept on 4C° with PBS (phosphate-buffered saline) + 0.1% BSA (bovine serum albumin) before staining. The cells were permeabilized with 0.1% Triton-X for 15 min, washed several times with PBS and TBS (Tris-buffered saline). After that, the samples were blocked with Background Sniper (Histolab Products AB, Sweden) for 10 min, rinsed with TBS several times and incubated with the primary antibody diluted in DaVinci Green Diluent (Histolab Products AB, Sweden) for 1 h at room temperature. Two different SLC2A1 antibodies binding to different epitopes were used as primary antibody specificity controls (ab32551 - rabbit polyclonal antibody to SLC2A1 from Abcam, dilution 1:200, and LS-C87465 – mouse polyclonal antibody to SLC2A1 from LifeSpan BioSciences, Inc., dilution 1:400). Secondary antibody and labeling controls were included. After washing with TBS, the samples were incubated with MACH3 Mouse or Rabbit Probe (depending on the primary antibody, Histolab Products AB, Sweden) for 12 min, rinsed with TBS and incubated with MACH3 Mouse or Rabbit M-Polymer HRP for 15 min in room temperature. The slides were rinsed again with TBS several times before incubation with Betazoid DAB (3, 3′-diaminobenzidine) Chromogen (Histolab Products AB, Sweden) for 5 min and washed with distilled water. The samples were subsequently counter stained with concentrated hematoxylin for 1 min and rinsed with tap water. Then the slides were dehydrated and mounted with Pertex before examination under microscope.

Samples from all 6 subjects were included in the evaluation. The stained slides were evaluated by three independent evaluators blinded for treatment and sample identity using the semi-quantitative manual scoring on a 5 point-scale: 0 (−), 1 (+/−), 2 (+), 3 (++) and 4 (+++). Conventional light microscopy at a magnification of 100x was used. Slides were evaluated for staining intensity and percentage of cells with each intensity. Because of the non-homogenic nature of the decidualization process, the four most decidualized areas from each slide were chosen for evaluation and photographed using image analysis system (Leica Imaging System Ltd., Cambridge, UK) before evaluation. The following equation was used to calculate the scores: 4 x percentage of strongly staining cells + 3 x percentage of moderately staining cells + 2 x percentage of weakly staining cells + 1 x percentage of very weakly staining cells, giving a range of 0 to 400. Scores were expressed as the highest value from the three observers and the highest score for each condition was used in statistical evaluation.

### Glucose uptake assay

Endometrial stromal cells were cultured and treated as described above using decidualizing agents and different concentrations of insulin. Before running the glucose uptake assay, the cells were starved in a serum- and insulin-free media containing 0.5 mM db-cAMP and 1 μM MPA for 4 hours. Glucose uptake was estimated by quantifying the cellular uptake of radioactive [3H]-2DG (1-[^3^H]-2-deoxy-D-glucose). The cells were incubated with [3H]-2DG containing glucose-free DMEM for 15 min, washed, solubilized with sodium dodecyl sulfate (SDS) and frozen over night before measuring the radioactivity of the samples in a liquid scintillation counter. Micro BCA™ protein assay kit (Thermo Fisher Scientific, USA) was used to quantify the amount of protein in each treatment well and glucose uptake was adjusted accordingly. All experiments were done at least in duplicate.

### Statistical analysis

Normality of data was tested with Shapiro-Wilk normality test. One-way ANOVA and Dunnett’s multiple comparisons test were used to analyze the effect of insulin on *SLC2A1* mRNA (messenger-RNA). Wilcoxon test was used to analyze the effect of decidualization on glucose uptake because the data was not normally distributed. For immunocytochemistry results we used a paired t-test since the data was normally distributed. A *p*-value of less than 0.05 was considered significant. To analyze the effect of decidualization on SLC2A1 gene expression, the effect of a FOXO inhibitor on *SLC2A1* gene expression, and the effect of insulin on glucose uptake, we adjusted the values of the control group (stromal cells or decidual cells) to 1 and calculated 95% confidence intervals (CI) to all other groups. A 95% CI that did not include the numerical value of the control group was considered statistically significant and marked with one asterisk (*) corresponding to the significance level of *p* < 0.05.

## Results

### Insulin downregulates *SLC2A1* mRNA level in decidualizing hESC

**Fig. 1:**
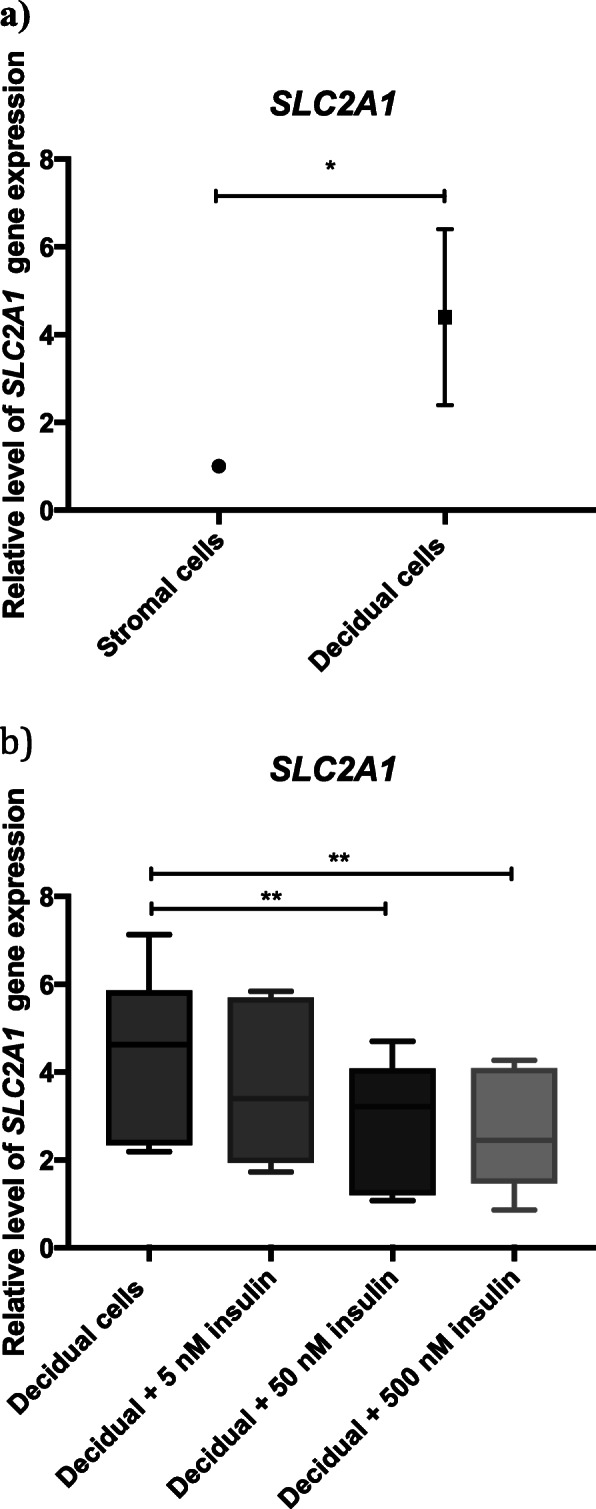
**a** Relative gene expression levels of *SLC2A1* in human endometrial stromal cells and decidualizing human endometrial stromal cells. The results are presented as mean values with 95% confidence interval (CI). Decidualizing endometrial stromal cells were compared to the control group (stromal cells) and differences considered statistically significant if 95% CI did not include the numerical value of the control group, in which case the significance was marked with one asterisk (*) corresponding to the significance level of *p* < 0.05. **b** Relative gene expression levels of *SLC2A1* in response to increasing concentrations of insulin in decidualizing human endometrial stromal cells. The values are presented as box plots showing minimum, first quartile, median, third quartile and maximum. One-way ANOVA and Dunnett’s multiple comparisons test was used for statistical analysis. ** = *p* < 0.01

Both prolactin and insulin like growth factor binding protein 1 (IGFBP-1), two of the most often used decidualization markers, increased significantly upon treating the endometrial stromal cells with decidualizing agents (data not shown). To study the effect of insulin on *SLC2A1* expression, we decidualized the hESC with and without different concentrations of insulin for 6 days and measured *SLC2A1* gene expression levels. We confirmed up-regulation of *SLC2A1* mRNA and protein expression during decidualization (Fig. 1a and Fig. 3a). Figure 1b shows the effect of increasing doses of insulin on the expression of *SLC2A1* mRNA. Treatment with 50 nM and 500 nM insulin resulted in significant dose-dependent down-regulation of *SLC2A1* expression, indicating that insulin is a regulator of *SLC2A1* gene expression at a transcriptional level in decidualizing hESC.

### FOXO1 inhibition downregulates the expression of *SLC2A1* mRNA in decidualizing hESC

**Fig. 2:**
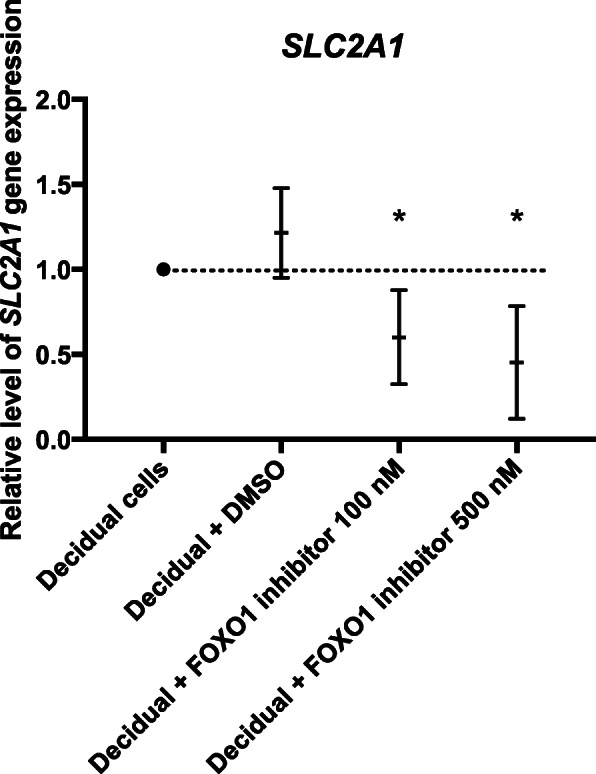
Relative gene expression levels of *SLC2A1* in decidualizing human endometrial stromal cells in response to treatment with a FOXO1 inhibitor. The results are presented as mean values with 95% confidence intervals (CI) for each culture condition. All groups were compared to the control group (decidual cells) and differences considered statistically significant if 95% CI did not include the numerical value of the control group, in which case the significance was marked with one asterisk (*) corresponding to the significance level of *p* < 0.05

Insulin transcriptionally inactivates FOXO1 in decidualizing hESC thereby affecting the expression of FOXO1 target genes [13]. To establish whether *SLC2A1* mRNA expression is regulated by FOXO1, we decidualized the cells in the presence of a FOXO1 inhibitor. The inhibition of FOXO1 by 100 nM and 500 nM of the inhibitor AS1842856 significantly decreased the expression of *SLC2A1* mRNA during decidualization (mean fold change 0.6 and 0.45, respectively), which indicates that *SLC2A1* is regulated by FOXO1 on a transcriptional level (Fig. 2).

### Insulin downregulates SLC2A1 protein level in decidualizing hESC

**Fig. 3:**
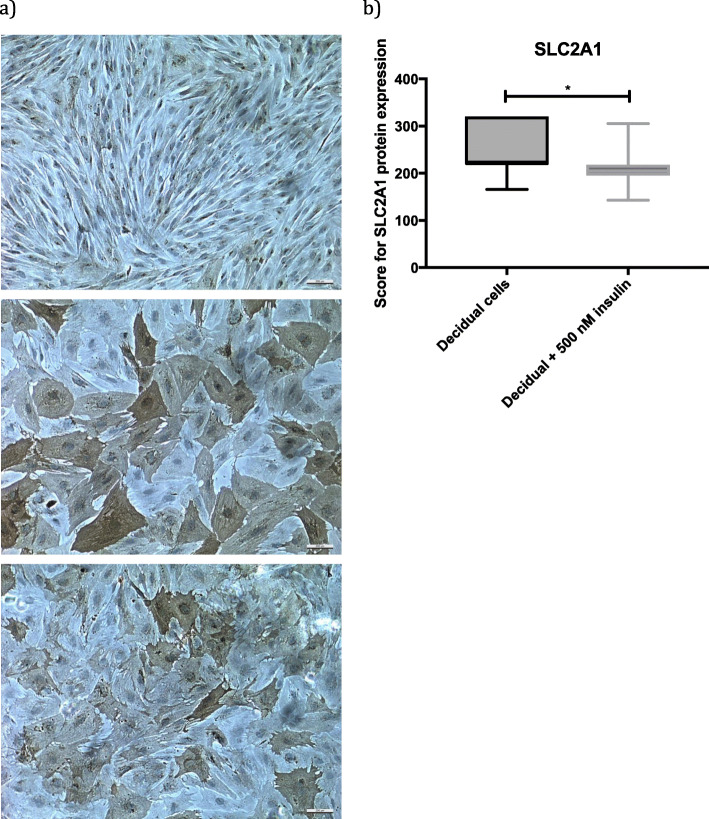
**a** Cytoplasmic staining of SLC2A1 protein in human endometrial stromal cells before and after decidualization with and without insulin. Top picture: human endometrial stromal cells, 100 x magnification. Center picture: decidualizing human endometrial stromal cells, 100 x magnification. Bottom picture: human endometrial stromal cells decidualized in the presence of 500 nM insulin, 100 x magnification. **b** Relative levels of SLC2A1 protein expression in response to insulin treatment. The values are presented as box plots showing minimum, first quartile, median, third quartile and maximum. Paired t-test was used for statistical analysis. *p* < 0.05 was considered significant. * = *p* < 0.05

To evaluate the effect of insulin on SLC2A1 protein expression we decidualized hESC with and without 500 nM insulin for 6 days and quantified SLC2A1 protein levels using immunocytochemistry (Fig. 3a). Secondary antibody and labeling controls did not show any non-specific staining. Two different antibodies binding to different epitopes of SLC2A1 protein were used and both resulted in similar staining patterns. We could show a slight but significant down-regulation of SLC2A1 protein expression in insulin-treated cells (Fig. 3a, b).

### Insulin decreases glucose uptake in decidualizing hESC

**Fig. 4:**
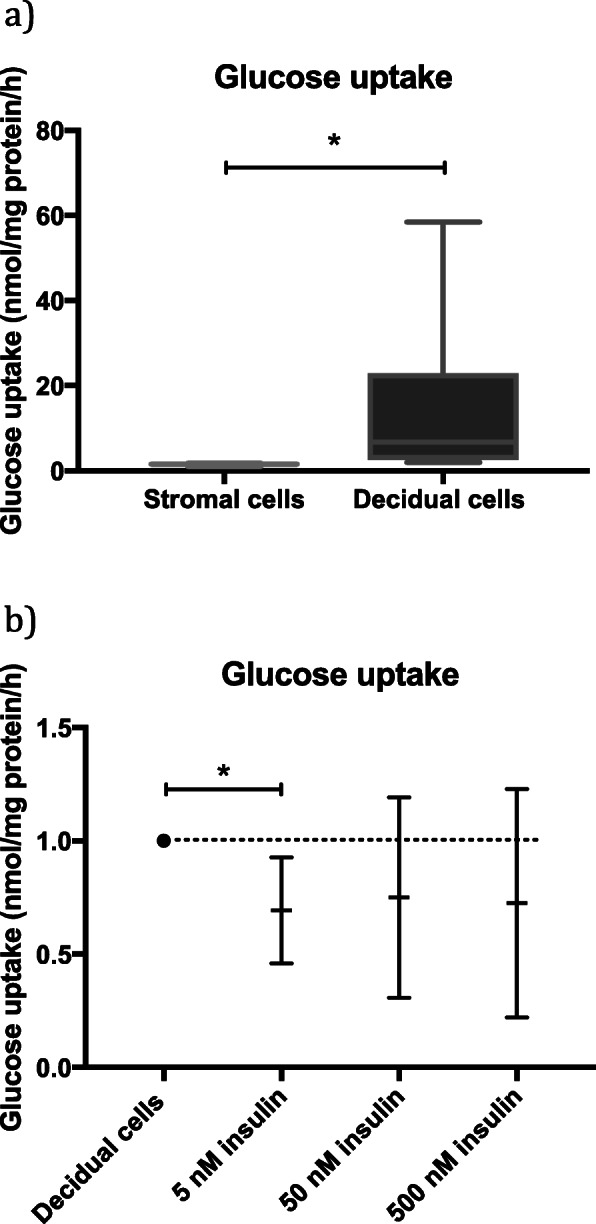
**a** Relative levels of glucose uptake in non-decidualized and decidualized human endometrial stromal cells. The values are presented as box plots showing minimum, first quartile, median, third quartile and maximum. Wilcoxon test was used for statistical analysis. *p* < 0.05 was considered significant. * = *p* < 0.05. **b** Relative levels of glucose uptake in decidualized human endometrial stromal cells in response to different concentrations of insulin. The results are presented as mean values with 95% confidence intervals (CI) for each culture condition. All groups were compared to the control group (decidual cells) and differences considered statistically significant if 95% CI did not include the numerical value of the control group in which case the significance was marked with one asterisk (*) corresponding to the significance level of *p* < 0.05

We measured glucose uptake in both non-decidualized and decidualized endometrial stromal cells and found a significant increase in glucose uptake after decidualization by a median fold change of 4.2 (Fig. 4a). We studied the effect of different concentrations of insulin on glucose uptake in decidualized hESC. In response to insulin, the median level of glucose uptake was lower in all groups but significantly decreased only after treatment with 5 nM insulin (mean fold change 0.69; 95% CI of 0.46 to 0.93) (Fig. 4b).

## Discussion

SLC2A1 expression is upregulated during decidualization, whereas insulin could have a negative impact on the same process. The present study is the first to show that high doses of insulin downregulate SLC2A1 mRNA and protein levels during decidualization of human endometrial stromal cells. The downregulation of SLC2A1 appears to be at least partly mediated by transcriptional inactivation of *FOXO1*. Decidualization was associated with increased glucose uptake, whereas insulin treatment resulted in a slight inhibition of the glucose uptake, although not significant for all insulin concentrations.

The insulin-mediated glucose uptake by solute carrier family 2 member 4 (SLC2A4:previously known as GLUT4) has been studied in many tissues [17]. However, there is data suggesting that insulin also has an effect on SLC2A1-mediated glucose uptake [8, 18]. SLC2A1 is the most common glucose transporter in human endometrium [6]. We have previously demonstrated that weight loss in obese women with PCOS, resulted in reduced circulating insulin levels, concomitantly enhanced insulin signaling and increased SLC2A1 expression in the endometrium [16]. Furthermore, an in vitro study from our research group suggested that high insulin levels may have a negative impact on the decidual transcriptome by transcriptional inactivation of FOXO1 [13]. We therefore hypothesized that SLC2A1 and insulin might interact in glucose uptake regulation and decidualization of human endometrium.

Indeed, the present study showed that high doses of insulin suppressed the expression of both gene and protein levels of SLC2A1 in decidualizing hESC. To the best of our knowledge, this is the first study on SLC2A1 regulation by insulin in decidualizing endometrial stromal cells derived from experiments using insulin-treated primary endometrial stromal cells. Furthermore, we confirmed for the first time that *SLC2A1* is a target of FOXO1 [19], an important transcription factor during decidualization. Since insulin is known to affect FOXO1 target genes via transcriptional inhibition [13], the downregulation of SLC2A1 by insulin seems at least partly be mediated by FOXO1.

Decidualization is proposed to be a highly energy-dependent process resulting in increased cellular glycogen levels [5]. SLC2A1 expression is upregulated and accompanied by an increase in glucose uptake during decidualization [10, 11]. Despite its upregulation upon decidualization, SLC2A1 is not considered as a classical marker of decidualization as it is abundantly expressed in undifferentiated stromal cells, too. Here, we confirm that glucose uptake increases upon decidualization of human endometrial stromal cells [11]. Since SLC2A1 is the dominating glucose transporter in endometrial stromal cells [12], we expected that decreased SLC2A1 expression in response to insulin would result in impaired glucose uptake. In support, we found a slight decrease of glucose uptake by insulin, although not consistent for all insulin concentrations. This is indirect evidence that the effect of insulin on glucose uptake is medicated by SLC2A1. However, we cannot exclude that other glucose transporters also could be involved.

It is unclear how high insulin affects the complex nature of decidualization since it does not seem to affect the morphological changes induced by decidualization. However, we have previously demonstrated extensive changes in the transcription profile of decidualizing endometrial stromal cells by high insulin indicating dysregulation of the process [13]. Furthermore, a recent study by Neff et al. demonstrated that loss of insulin receptor substrate 2 (IRS2), a signaling molecule that mediates the effect of insulin, suppressed the expression of decidualization markers and SLC2A1, and decreased the glucose uptake of the cells. They also showed that high insulin doses caused down-regulation of the insulin receptor and certain decidualization markers, which is in line with our previous publication [11, 13]. In the present study, we demonstrate the direct downregulating effect of high doses of insulin on SLC2A1 expression and glucose uptake. These data support the role of insulin in dysregulation of decidualization.

Although the present study is an experimental in vitro investigation, the results may have implications for clinical conditions like PCOS and obesity. These hyperinsulinemic disorders are associated with anovulation but also endometrial dysfunction as reflected by impaired decidualization and implantation, increased risk of miscarriage and preeclampsia [20–22]. On the other hand, lifestyle intervention resulting in weight loss and enhanced insulin sensitivity, improves reproductive function in these women [16, 23]. Normalized endometrial function and decidualization could be involved in this improvement. Our study provides information on the multifactorial effect of insulin on endometrial decidualization and the regulation of glucose homeostasis. Increased knowledge of molecular mechanisms behind reproductive disorders associated with hyperinsulinemia might lead to new therapeutic approaches in the treatment of infertility.

The limitations of the study include restricted number of endometrial samples and the interindividual variation of the results. The women had regular menstrual cycles but not all had been pregnant and therefore normal fertility was not proven. Also, one of the subjects was overweight with a BMI of 28. However, we consider the use of primary endometrial stromal cells instead of a cell line a strength of the study. Insulin concentrations were chosen based on previous studies [24–27]. Tissue concentrations of insulin have been shown to vary in different tissues and are not directly related to circulating levels [28]. The normal variation of insulin concentration in the endometrium is not known.

## Conclusions

In conclusion, this study provides a further piece of understanding of the complex process of decidualization. We showed that high concentrations of insulin downregulate both gene and protein expression of SLC2A1 accompanied by a slight decrease in glucose uptake in decidualizing human endometrial stromal cells. Although our results show that SLC2A1 is downregulated by increased insulin, we cannot fully conclude the functional role of SLC2A1 for decidualization as this was not the specific aim of the study. Furthermore, clinical studies are needed to determine the importance of insulin regulation of endometrial function and decidualization in patient groups with insulin resistance and hyperinsulinemia.

## Data Availability

The datasets used and analyzed during the current study are available from the corresponding author on reasonable request.

## Abbreviations

[3H]-2DG: 1-[^3^H]-2-deoxy-D-glucose
BSA: Bovine serum albumin
CI: Confidence interval
DAB: 3, 3′-diaminobenzidine
db-cAMP: N^6^, 2`-O-dibutyryladenosine cAMP
FOXO1: Forkhead box O1
hESC: Human endometrial stromal cells
IRS2: Insulin receptor substrate 2
MPA: Medroxyprogesterone-17-acetate
mRNA: Messenger-RNA
PBS: Phosphate-buffered saline
PCOS: Polycystic ovary syndrome
SDS: Sodium dodecyl sulfate
SLC2A1: Solute carrier family 2 member 1
SLC2A4: Solute carrier family 2 member 4
TBS: Tris-buffered saline
